# Effect of Silver/Reduced Graphene Oxide@Titanium Dioxide (Ag/rGO@TiO_2_) Nanocomposites on the Mechanical Characteristics and Biocompatibility of Poly(Styrene-*co*-Methyl Methacrylate)-Based Bone Cement

**DOI:** 10.3390/polym17141970

**Published:** 2025-07-18

**Authors:** Mohan Raj Krishnan, Reem M. Alshabib, Edreese H. Alsharaeh

**Affiliations:** College of Science and General Studies, Alfaisal University, P.O. Box 50927, Riyadh 11533, Saudi Arabia; mkrishnan@alfaisal.edu (M.R.K.); ralshabib@alfaisal.edu (R.M.A.)

**Keywords:** acrylic bone cement, copolymer, nanocomposites, reduced graphene oxide, biocompatibility

## Abstract

This study reports the impact of a silver nanoparticles/reduced graphene oxide@titanium dioxide nanocomposite (Ag/rGO@TiO_2_) on the mechanical and biocompatibility properties of poly(styrene-co-methylmethacrylate)/poly methyl methacrylate (PS-PMMA/PMMA)-based bone cement. The chemical, structural, mechanical, and thermal characteristics of Ag/rGO@TiO_2_ nanocomposite-reinforced PS-PMMA bone cement ((Ag/rGO@TiO_2_)/(PS-PMMA)/PMMA) were evaluated using Fourier Transform Infrared spectroscopy (FT-IR), X-ray diffraction (XRD), nano-indentation, and electron microscopy. FT-IR, XRD, and transmission electron microscopy results confirmed the successful synthesis of the nanocomposite and the nanocomposite-incorporated bone cement. The elastic modulus (E) and hardness (H) of the ((Ag/rGO@TiO_2_)/(PS-PMMA)/PMMA) bone cement were measured to be 5.09 GPa and 0.202 GPa, respectively, compared to the commercial counterparts, which exhibited E and H values of 1.7 GPa to 3.7 GPa and 0.174 GPa, respectively. Incorporating Ag/rGO@TiO_2_ nanocomposites significantly enhanced the thermal properties of the bone cement. Additionally, in vitro studies demonstrated that the bone cement was non-toxic to the MG63 cell line.

## 1. Introduction

Natural bone possesses an impressive compressive strength, reaching up to 170 MPa, but its tensile strength, ranging from 104 to 121 MPa, and shear stress strength at 51.6 MPa are relatively low [1,2]. This disparity indicates that bones are susceptible to fractures, particularly when subjected to torsional forces [3,4]. In essence, bones are more vulnerable to tension or twisting than they are to compressive forces [5,6]. Although bones exhibit a certain degree of brittleness, comprising approximately 80% calcium phosphate, they also demonstrate remarkable elasticity, primarily due to the presence of collagen, which provides some flexibility [7]. As individuals age, the likelihood of bone failure increases significantly due to various factors, including mechanical stress, injuries, diseases, infections, and tumors. Conditions, such as osteoporosis, scoliosis, and osteomyelitis, can substantially weaken the structural integrity of bones, making them more prone to fractures or deterioration from even slight movements or stress [8]. Decades ago, researchers discovered an innovative technique involving the mixing of methyl methacrylate (MMA) liquid with polymethylmethacrylate (PMMA) powder, along with benzoyl peroxide (BPO) [9,10]. This combination forms a pliable, dough-like substance that, once applied, undergoes a polymerization process to harden into a resilient material [11,12]. This advancement has significantly impacted the field of orthopedic surgery and bone repair, showcasing the interplay between biomaterials and natural bone healing [13,14,15]. Initially, the versatile mixtures of polymethyl methacrylate (PMMA) were primarily utilized to treat cranial defects, marking a significant advancement in surgical practices [16]. Over time, their applications have evolved remarkably, extending into the realms of denture bases and various prosthetic materials [17,18,19,20,21]. This expansion can be attributed to their exceptional strength, remarkable stability, widespread commercial availability, and affordability [22]. Furthermore, it has been discovered that these mixtures can be molded into solid forms of desired shapes using an array of plaster molds, showcasing their adaptability [23]. As the years progressed, enhancements in the preparation methods and surface attributes of PMMA were achieved through the use of optimized molds and the incorporation of spherical polymer particles, resulting in superior results [24,25].

Acrylic bone cement, commonly referred to as PMMA bone cement, is composed of a solid element, which is the PMMA powder, paired with an initiator known as benzoyl peroxide (BPO), along with a liquid component—methyl methacrylate (MMA)—which is activated by a room-temperature agent called dimethyl paratoluidine (DMPT) [11,26]. When these solid and liquid components are carefully mixed, they initially form a dough-like consistency that gradually transitions into a robust polymer as the curing process unfolds [15]. The clinical success of acrylic bone cement is influenced by a myriad of factors, including incorporating various additives, such as antibiotics and radio pacifiers, the mixing technique employed, the specific chemical composition, and the biocompatibility of each component [27]. For example, innovative formulations incorporating nanostructured carbon materials, such as carbon nanotubes [28,29], graphene [30,31], and graphene oxide [32], can be utilized to enhance bone cement’s mechanical and biocompatibility properties significantly [33,34]. Additionally, the integration of silver (Ag) nanoparticles into the bone cement imparts antimicrobial properties, thereby addressing infection concerns that may arise post-surgery [35]. Developing polymer nanocomposite-based bone cement presents a promising solution to address certain inherent limitations associated with traditional acrylic-based bone cements. These multifaceted enhancements underline the persistent and evolving nature of acrylic bone cement formulations, which seek optimal clinical application performance [16].

Incorporating TiO_2_ nanoparticles into bone cement can enhance mechanical properties, such as compressive and flexural strength, as well as fatigue resistance, thereby improving structural integrity and reducing fracture risk [36,37]. TiO_2_ nanoparticles also exhibit antimicrobial properties, which can help prevent infections at the implant site, potentially improving patient outcomes [38,39,40]. Additionally, they can act as carriers for therapeutic agents [41,42], enabling the controlled release of antibiotics or growth factors to promote bone healing and prevent infection. TiO_2_ nanoparticles can be integrated into composite scaffolds or bioactive coatings for tissue engineering [43,44]. When combined with biocompatible polymers or ceramics, they enable the creation of customized implants for specific applications, such as bone defect repair or joint replacement [45,46,47]. Understanding their degradation behavior in biological environments is crucial for long-term stability and biocompatibility. While TiO_2_ is generally biostable, more research is needed on its long-term interactions with tissues. Overall, TiO_2_ nanoparticles enhance the performance of bone cement in orthopedic and dental applications due to their biocompatibility, mechanical reinforcement, radiopacity, antimicrobial properties, and potential for controlled drug delivery, making them valuable for advanced biomaterials in bone regeneration [48,49,50].

Therefore, our objective is to formulate a bone cement material utilizing Ag/rGO@TiO_2_ nanocomposites integrated into a polystyrene-polymethyl methacrylate (PS-PMMA) copolymer, positioning it as an improved and multifunctional bone cement. A recent study by Liu et al. demonstrated that Ag@3D-TiO_2_ scaffolds exhibited sustained antimicrobial activity, enhanced cell proliferation, and promoted osteogenic differentiation, resulting in increased extracellular matrix mineralization. Also, in vivo studies showed anti-inflammatory effects with significant fibrous connective tissue around the scaffolds and enhanced biocompatibility compared to 3D-Ti and 3D-TiO_2_ [51]. Similarly, Yao et al. demonstrated that silver-loaded TiO_2_ nanotube arrays exhibit enhanced long-term antibacterial and osteogenic properties [52]. Therefore, incorporating TiO_2_ along with Ag/rGO nanocomposites could significantly improve the mechanical strength and biocompatibility of the acrylic bone cement formulations. This advanced cement formulation can also offer valuable functionalities, including antimicrobial properties attributable to silver and TiO_2_ nanoparticles. These enhancements could significantly elevate the performance and effectiveness of bone cement in medical applications, paving the way for better patient outcomes.

## 2. Experimental

### 2.1. Chemicals

Styrene and methyl methacrylate monomers (purity > 99%) were acquired from Sigma-Aldrich, St. Louis, MO, USA. Azoisobutyronitrile (AIBN, Mw = 164.21 g/mol) was sourced from Aldrich and subsequently recrystallized using methanol. Benzoyl peroxide (BPO, synthetic grade with an assay of 72.0–80.0%) was also purchased from Sigma-Aldrich. A titanium (III) chloride solution (10–15% TiCl_3_ basis) was obtained from Sigma-Aldrich as well. Cetyltrimethylammonium bromide (CTAB, 99%) was acquired from Sisco Research Laboratories (SRL). Hydrazine hydrate solution (80% in water, intended for synthesis) and ammonia solution (28–30%, for analysis, EMSURE^®^ ACS Reag. Ph Eur) were purchased from Merck. The monomers and other chemicals were used as received. The reduced graphene oxide (rGO) was synthesized using a modified Hummers’ method. Details on the preparation of rGO and Ag/rGO can be found in our previous works [53,54,55,56].

### 2.2. Preparation of Ag/rGO@TiO_2_

The Ag/rGO@TiO_2_ nanocomposite was synthesized through a one-pot sol-gel method. To prepare the sample, 250 mg of CTAB was added to 250 mL of a 1:1 mixture of ethanol and deionized water, ensuring that it was well dissolved. Next, 5.5 mg of AgNO_3_ was introduced and stirred until it was fully dissolved. Following this, 14.8 mg of GO was added and dispersed via ultrasonication. Then, 11.7 mL of TiCl_3_ solution was added to the mixture dropwise, resulting in the formation of a yellow precipitate. A suitable amount of ammonia solution (approximately 1.6 M, 150 mL) was then added to neutralize the acidic solution. The mixture was subsequently exposed to microwave radiation for 45 min. Afterward, the solution was filtered to isolate the nanocomposite, which was then washed with 200 mL of absolute ethanol and 500 mL of distilled water. Following the washing process, the nanocomposite was dried at 80 °C for 10 h in a drying oven. Finally, the nanocomposite was calcined in a box furnace (NaberTherm, Lilienthal, Germany) at 500 °C for 4 h, with a heating ramp of 1 °C/min.

### 2.3. Preparation of (Ag/rGO@TiO_2_)/(PS-PMMA) Nanocomposite

To prepare the (Ag/rGO@TiO_2_)/(PS-PMMA) nanocomposite, a quantity of 2.0 wt.% of Ag/rGO@TiO_2_ relative to the PS-PMMA copolymer was accurately weighed and thoroughly mixed with a co-monomer mixture of S and MMA in a 1:1 weight ratio, along with 0.01 wt.% of the initiator AIBN. The mixture was then sonicated for 30 min at room temperature to ensure complete homogenization of the components. Following this, the temperature was raised to 70 °C to initiate the polymerization process. Under the heating conditions, AIBN decomposed into two free radicals, which in turn generated co-monomer radicals, leading to the copolymerization of the monomers. As a result, the (Ag/rGO@TiO_2_)/(PS-PMMA) nanocomposite was formed. The polymerization was allowed to proceed for five days, after which the resulting nanocomposite was washed multiple times with hot ethanol at 50 °C to eliminate any homopolymers or oligomers that could be present. The washed nanocomposites were then dried at 40 °C. It should be noted that the half-life of AIBN is approximately 5 h at 70 °C, i.e., 99.99% of the AIBN is decomposed in <3 days. We typically extended the duration of the polymerization beyond this time frame to ensure that the reaction reached full completion, allowing for the thorough formation of the polymer composite while minimizing the unreacted monomers and oligomers. This additional step was aimed at enhancing the overall yield and efficacy of the final product.

### 2.4. Ball-Milling of (Ag/rGO@TiO_2_)/(PS-PMMA) Nanocomposite

A SPEX Sample Prep 8000M Mixer/Mill (SPEX SamplePrep, Metuchen, NJ, USA), a high-energy ball milling device, was used to ball mill the samples. The (Ag/rGO@TiO_2_)/(PS-PMMA) nanocomposite sample was ball-milled for four hours to prepare the bone cement solid component. For a batch of sample preparation, 7 g of polymer nanocomposite sample was used and milled with two 12.7 mm stainless steel balls and four 6.35 mm stainless steel balls.

### 2.5. Preparation of the (Ag/rGO@TiO_2_)/(PS-PMMA)/PMMA Bone Cement

A typical bone cement device comprises both solid and liquid components. The solid component consists of a nanocomposite powder made from (Ag/rGO@TiO_2_)/(PS-PMMA) and a specific quantity of BPO initiator. The liquid component is formulated with MMA monomer, N, N-dimethyl p-toluidine (DMPT), and hydroquinone (HQ) to prevent the auto-initiation of MMA. The BPO/DMPT is known as a redox initiation system wherein the DMPT acts as a reducing agent. In this study, we utilized (Ag/rGO@TiO_2_)/(PS-PMMA) nanocomposite powder as the solid component, while the liquid component consisted of a mixture of MMA and 50 µL of DMPT. The solid-to-liquid ratio was maintained at a 2:1 weight-to-weight (*w*/*w*) ratio. The solid and liquid components were thoroughly mixed to create the final bone cement formulation (Ag/rGO@TiO_2_)/(PS-PMMA)/PMMA.

### 2.6. Characterizations

#### 2.6.1. Fourier Transform-Infrared Spectroscopy (FT-IR)

The FT-IR spectra of the samples were recorded using the Thermo Scientific Nicolet-iS10 instrument (Thermo Fisher Scientific, Waltham, MA, USA). KBr was used to prepare the samples, and the measurements were conducted in transmission mode. The spectra were obtained in the wavenumber range of 4000 to 500 cm^−1^.

#### 2.6.2. X-Ray Diffraction (XRD)

The Rigaku MiniFlex 600 (Rigaku Corporation, Tokyo, Japan) was used to record the XRD of the samples, employing Cu radiation at 40 kV and 15 mA with a Cu Kα wavelength of 1.54 Å. The analysis was conducted over a 5° to 80° range.

#### 2.6.3. Nanoindentation Tests

The NanoTest™ system (Micro Materials, Wales, UK) was utilized to measure the elastic modulus (E) and hardness (H) of the samples. Nanoindentation studies were performed using a NanoTest™ system (Micro Materials, Wales, UK) equipped with a diamond Berkovich indenter. Each loading and unloading cycle lasted 10 s, with a dwell time of 5 s at each peak load. The force-displacement (P-h) profile was utilized to determine the hardness (H) and elastic modulus (Young’s modulus, E) of the samples. For the indenter, the elastic modulus (E_i_) and Poisson’s ratio (ν_i_) were taken as 1140 GPa and 0.07, respectively [57], while the Poisson’s ratio (νs) of the sample was set at 0.33. During measurement, the samples were mounted onto a steel disc substrate using cyanoacrylate adhesive.

#### 2.6.4. High-Resolution Transmission Electron Microscopy (HR-TEM)

High-resolution transmission electron microscopy (HR-TEM) images of the samples were captured using the HT7800, which was operated at an accelerating voltage of 120 kV. To prepare the samples for imaging, thin flakes with a thickness of less than 80 nm were carefully cut using an ultra-microtome. These samples were then placed onto a carbon-coated copper grid to prevent charging effects. This meticulous preparation ensures high-quality imaging and accurate analysis of the sample’s structural characteristics.

### 2.7. In Vitro Studies

#### 2.7.1. Cell-Culturing

MG63 cells were cultured in a T75 tissue culture flask, undergoing eight passages to ensure optimal growth and stability. This process involved carefully monitoring cell density and morphology to maintain healthy cell characteristics at each passage. As a result, we successfully established a cell bank comprising 28 vials, each containing aliquots of actively growing MG63 cells. These vials were securely stored at low temperatures to preserve the viability and integrity of the cells for experimental use.

#### 2.7.2. Passaging

The media, consisting of DMEM (Dulbecco’s Modified Eagle Medium) supplemented with 10% FBS (fetal bovine serum), 1% penicillin-streptomycin (P/S), and 1% L-glutamate, along with 0.25% trypsin and PBS (phosphate-buffered saline), was placed in a water bath for 30 min. The cell culture hood was cleaned for one minute before use. The media in the flask was aspirated, and the flask was washed three times with PBS. Ten percent trypsin was added, and the flask was incubated at 37 °C for 5 min. Afterward, 80% media was added, and the mixture was transferred to a centrifuge tube and centrifuged at 100 XG for eight minutes. The supernatant was aspirated, fresh media were added, and the contents were mixed. Finally, they were transferred to two new flasks and incubated at 37 °C in humidified air with 5% CO_2_.

#### 2.7.3. Alamar Blue Assay

MG63 cells were seeded at 0.01 × 10^6^ cells/mL in a 96-well plate and treated with 2 mg/mL of nanomaterial (*n* = 3). A 10% Alamar Blue solution in phenol red-free DMEM was added, and the absorbance was measured using an Epoch BioTek spectrophotometer (Winooski, VT, USA) at an excitation of 570 nm and an emission of 600 nm, starting 4 h post-treatment. Measurements were taken at three initial time points, followed by five additional points at 24, 48, 72, 144, and 168 h.

## 3. Results and Discussion

### 3.1. Silver/Reduced Graphene Oxide@Titanium Dioxide/(Poly(Styrene-co-Methyl Methacrylate)/Polymethyl Methacrylate) ((Ag/rGO@TiO_2_)/(PS-PMMA)/PMMA) Nanocomposite Bone Cement

The (Ag/rGO@TiO_2_)/(PS-PMMA)/PMMA bone cement sample was prepared by combining the solid component of Ag/rGO@TiO_2_)/(PS-PMMA) with BPO (initiator) and the liquid component, consisting of a mixture of MMA and DMPT (co-initiator or cold curing agent). At room temperature, when DMPT interacts with BPO molecules, it facilitates a redox reaction that generates free radicals of BPO (I^•^). These initiator free radicals then convert MMA molecules into free radicals as well. Once the MMA free radicals (I-MMA^•^) are produced, they react randomly with the MMA, leading to chain propagation (I-(MMA)_n_^•^). The polymerization reaction concludes following chain propagation, either through disproportionation (MMA)_m_ or via a coupling reaction (MMA)_2m_ of the polymeric chain radicals. Furthermore, the nanofiller Ag/rGO@TiO_2_ contains chemically reactive linkages of rGO (-C=C-), which actively participate in the ongoing polymerization, resulting in the formation of the (Ag/rGO@TiO_2_)/(PS-PMMA)/PMMA) nanocomposite. The detailed curing mechanism of the bone cement can be found in our previous works [33,35].

### 3.2. FT-IR

**Figure 1:**
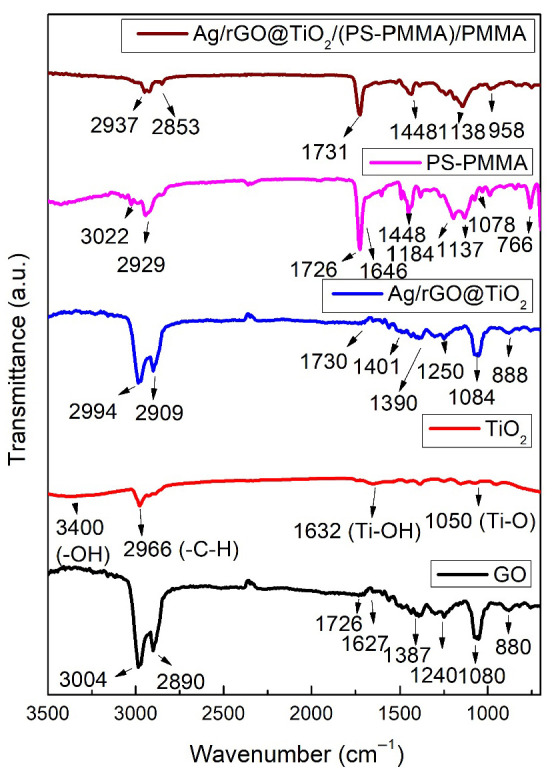
FT-IR spectra of GO, TiO_2_ nanoparticles, Ag/rGO@TiO_2_ nanofiller, PS-PMMA copolymer, and Ag/rGO@TiO_2_/(PS-PMMA)/PMMA bone cement.

Figure 1 shows the FT-IR spectra of graphene oxide (GO), titanium dioxide (TiO_2_) nanoparticles, silver/reduced graphene oxide@TiO_2_ (Ag/rGO@TiO_2_) nanofiller, polystyrene-polymethyl methacrylate (PS-PMMA) copolymer, and Ag/rGO@TiO_2_/(PS-PMMA)/PMMA bone cement. The GO demonstrates a range of vibrational modes indicative of its functional groups. Notably, the epoxide groups (C–O–C) are reflected by peaks in the region of 1230–1320 cm^−1^. Additionally, vibrational modes associated with sp^2^-hybridized carbon-carbon double bonds (C=C) appear between 1500 and 1600 cm^−1^, revealing in-plane vibrations that can be attributed to the aromatic structure of the material. The presence of carboxyl functional groups (COOH) is evidenced by multiple peaks in the range of 1650–1750 cm^−1^, which include the C–O stretching vibration at a wavenumber of 1080 cm^−1^. Moreover, vibrational signatures of carbonyl species (C=O) can be distinguished by absorption bands between 1600–1650 cm^−1^ as well as in the higher range of 1750–1850 cm^−1^. Hydroxyl groups (–OH) are identified through broad peaks spanning from 3050 to 3800 cm^−1^. In the context of titanium dioxide (TiO_2_) nanoparticles, the formation of Ti–O bonds is substantiated by a peak observed at 1050 cm_−1_, indicating successful bonding interactions. The hydroxyl functionality present on the synthesized TiO_2_ nanoparticles is corroborated by the band appearing at around 3400 cm^−1^. Furthermore, a peak at 1632 cm^−1^ provides evidence of hydroxyl groups at the surface level, characterized as Ti–OH bonds among the TiO_2_ particles. An additional band located at 2966 cm^−1^ is attributed to the asymmetric stretching of C–H groups present within the nanoparticle matrix. In the case of the Ag/rGO@TiO_2_ nanocomposites, several distinctive vibrational features can be noted. A peak at 888 cm^−1^ corresponds to the stretching of the –C–O bond, while the peak at 1084 cm^−1^ signifies the presence of C–O–C linkages. The band observed at 1250 cm^−1^ is associated with –C–OH groups, and a prominent band at 2994 cm^−1^ indicates the hydroxyl (–OH) functional groups integrated into the composite structure, illustrating a synergistic interaction among its constituents. The PS-PMMA copolymer exhibited various characteristic peaks. Notably, the peak at 711 cm^−1^ is attributed to the in-phase bending vibration of the mono-substituted ring in polystyrene (PS). The peak observed at 766 cm^−1^ corresponds to the asymmetric stretching band of the C–C–O group in polymethyl methacrylate (PMMA). Additionally, a peak at 1078 cm^−1^ is observed, representing the stretching mode of the C–O–C bond. The bending mode of the methyl groups (–CH_3_) is evidenced by the peaks at 1444 cm^−1^ and 2929 cm^−1^. Furthermore, the stretching mode of the C=C bond in PS is noted at 1646 cm^−1^, and the peak at 1726 cm^−1^ signifies the presence of the carbonyl (C=O) group in PMMA.

The Ag/rGO@TiO_2_/(PS-PMMA)/PMMA bone cement showed various characteristic peaks corresponding to its primary components, namely the PS-PMMA matrix and the Ag/rGO@TiO_2_ nanofiller. Notably, these peaks exhibited significant shifts from their original positions, a crucial indicator of the interactions occurring within the composite material. The observed relative shifts in the peaks not only highlight the successful incorporation of Ag/rGO@TiO_2_ into the PS-PMMA matrix but also confirm the effective formation of Ag/rGO@TiO_2_/(PS-PMMA)/PMMA nanocomposites.

### 3.3. XRD

**Figure 2:**
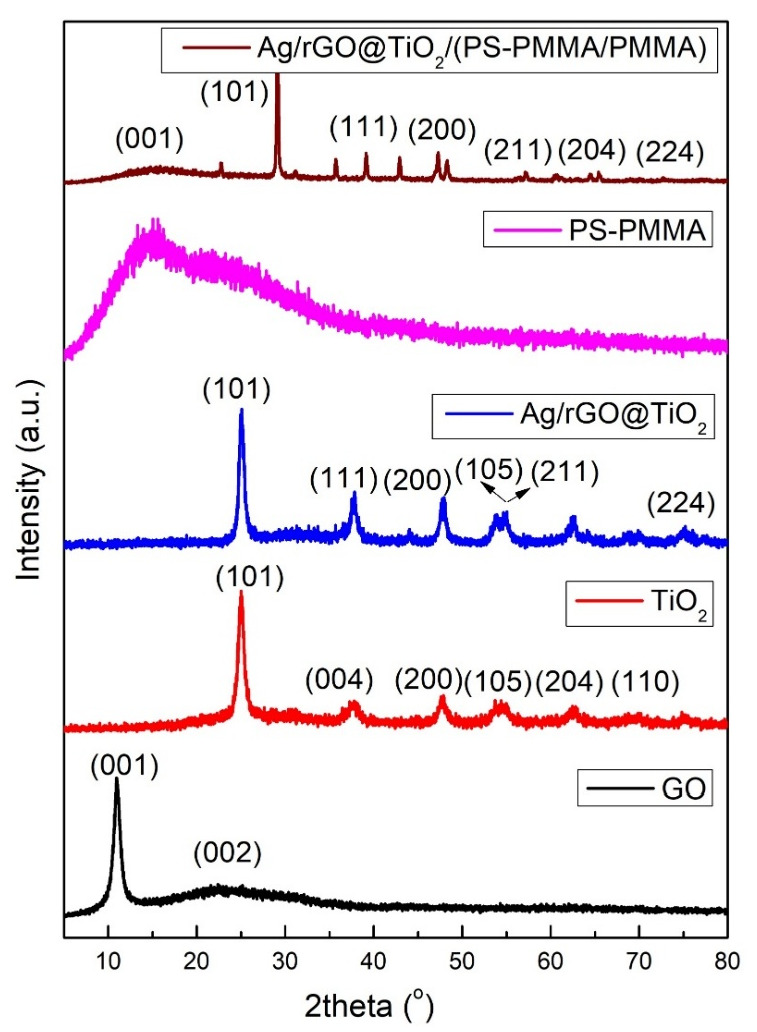
XRD patterns of GO, TiO_2_ nanoparticles, Ag/rGO@TiO_2_ nanofiller, PS-PMMA copolymer, and Ag/rGO@TiO_2_/(PS-PMMA)/PMMA bone cement.

Figure 2 shows the XRD patterns of GO, TiO_2_ nanoparticles, Ag/rGO@TiO_2_ nanofiller, PS-PMMA, and Ag/rGO@TiO_2_/(PS-PMMA)/PMMA bone cement. The XRD pattern of GO revealed two prominent characteristic peaks at 10.9° (002) and 25.8° (001), indicating its layered structure and crystallinity. In the case of TiO_2_ nanoparticles, distinct peaks were observed at 25.3° (101), 37.9° (004), 48.0° (200), 54.4° (105), 57.3° (205), and 62.8° (110), confirming the successful synthesis and crystallization of the TiO_2_ in the anatase phase. Furthermore, the Ag/rGO@TiO_2_ nanofiller exhibited several diffraction peaks at 25.3° (101), 37.9° (004), 48.0° (200), 54.4° (105), 63.8° (211), and 66.2° (224), which also validated the successful formation of this composite material. In contrast, the PS-PMMA copolymer displayed an absence of significant peaks, supporting the notion that it is an entirely amorphous material, which is typical for such copolymers. The XRD pattern of the Ag/rGO@TiO_2_/(PS-PMMA)/PMMA bone cement material revealed a pronounced and sharp peak at 25.3° (101) with considerable intensity, along with several other lower-intensity peaks that correlate with Ag/rGO@TiO_2_ components. This indicates that the composite structure is intact, suggesting effective integration of the nanofiller into the bone cement matrix. These comprehensive XRD results confirm the successful formation of the Ag/rGO@TiO2/(PS-PMMA)/PMMA bone cement. Moreover, the alignment of XRD findings with Fourier-transform infrared spectroscopy (FT-IR) results further substantiates the successful synthesis of the bone cement material.

### 3.4. Nanomechanical Properties

**Figure 3:**
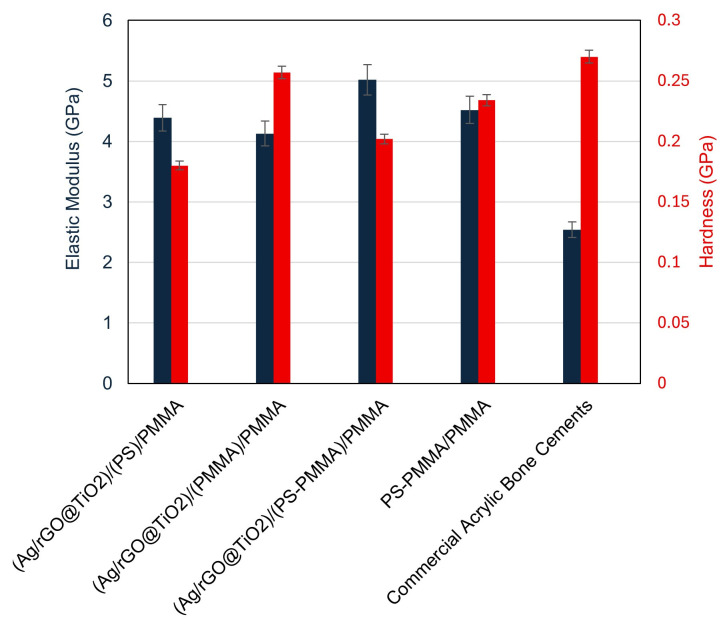
Nanomechanical properties of (Ag/rGO@TiO_2_)/(PS)/PMMA, (Ag/rGO@TiO_2_)/(PMMA)/PMMA, (Ag/rGO@TiO_2_)/(PS-PMMA)/PMMA, (PS-PMMA)/PMMA bone cements, and commercial acrylic bone cement.

Figure 3 shows a detailed analysis of the elastic modulus (E) and hardness (H) characteristics of different bone cement formulations, namely (Ag/rGO@TiO_2_)/(PS)/PMMA, (Ag/rGO@TiO_2_)/(PMMA)/PMMA, (Ag/rGO@TiO_2_)/(PS-PMMA)/PMMA, and (PS-PMMA)/PMMA. Among these, (PS-PMMA)/PMMA demonstrated an elastic modulus of 4.52 GPa and hardness of 0.23 GPa, while the (Ag/rGO@TiO_2_)/(PS-PMMA)/PMMA composite exhibited an elastic modulus of 5.09 GPa and a hardness of 0.202 GPa. Note that commercial acrylic bone cements (PMMA/MMA) have an elastic modulus in the range of 1.59 to 3.7 GPa. Examples of commercial acrylic bone cements and their E values include: Simplex^®^ P (Stryker) (E = 1.59–2.91 GPa), Osteopal^®^ G (Heraeus) (E = 1.74 GPa), Vertecem (Synthes) (E = 1.837 GPa), and KyphX^®^ HV-R™ (Kyphon) (E = 3.7 GPa) [58,59,60]. Incorporation of the PS segment to PMMA polymeric chains results in a significant enhancement in the elastic modulus value. Additionally, the incorporation of the Ag/rGO@TiO_2_ nanocomposite into the PS-PMMA/PMMA matrix resulted in a further enhancement of the elastic modulus of the composite bone cement. The significant enhancement in the elastic modulus of (Ag/rGO@TiO_2_)/(PS-PMMA)/PMMA (5.09 GPa) compared to that of commercial acrylic bone cement (1.59 to 3.7 GPa) suggests a potential for enhanced load-bearing capacity and improved structural integrity in the newly proposed bone cement. Additionally, a decrease in the hardness value of (Ag/rGO@TiO_2_)/(PS-PMMA)/PMMA (0.202 GPa) compared to that of PMMA/MMA cement (0.27 GPa) can be attributed to the newly formed interface of rGO nanosheets of the Ag/rGO@TiO_2_ with polymer PS-PMMA chains. The 2D-rGO nanosheets are likely to modify the interaction between the polymer chains, resulting in a more flexible and rigid nanocomposite.

To systematically assess the effect of PS and PMMA polymeric segments on the mechanical properties of their corresponding composites with Ag/rGO@TiO_2_, we prepared two distinct sample configurations: (Ag/rGO@TiO_2_)/(PS)/PMMA and (Ag/rGO@TiO_2_)/(PMMA)/PMMA. The E and H values were evaluated. The composite featuring the structure (Ag/rGO@TiO_2_)/(PS)/PMMA demonstrated impressive mechanical characteristics, with an elastic modulus of 4.39 GPa and a hardness value of 0.18 GPa. In contrast, the sample structured as (Ag/rGO@TiO_2_)/(PMMA)/PMMA exhibited slightly different properties, recording an elastic modulus of 4.13 GPa and a hardness of 0.257 GPa. These data reveal an intriguing relationship: while the incorporation of PMMA segments enhances the hardness of the composite, it simultaneously leads to a decline in its elastic modulus. Conversely, the presence of PS segments showcases the opposite effect, contributing to a reduction in hardness while promoting an increase in the elastic modulus. These findings offer valuable insights into the balancing act between the hardness and elasticity of polymeric composites in advanced material applications.

To systematically investigate the origin of the observed decrease in hardness and the concurrent increase in elasticity of the acrylic copolymer when integrated with the Ag/rGO@TiO_2_ nanofiller, two alternative bone cement formulations were developed. These formulations include (Ag/rGO@TiO_2_)/(PS)/PMMA and (Ag/rGO@TiO_2_)/(PMMA)/PMMA. The elastic modulus and hardness values obtained for the first formulation, (Ag/rGO@TiO_2_)/(PS)/PMMA, were found to be 4.39 GPa and 0.18 GPa, respectively. In comparison, the second formulation, (Ag/rGO@TiO_2_)/(PMMA)/PMMA, exhibited a slightly lower value of 4.13 GPa for the elastic modulus and an increased hardness of 0.22 GPa. Interestingly, the PS-based bone cement formulation exhibited a higher elasticity value and decreased hardness in comparison to that of the PMMA-based bone cement. i.e., increased weight fraction of the PMMA in the bone cement, resulting in a higher hardness with lower elasticity values, and the increased weight fraction of PS leads to higher elasticity with lower hardness. This highlights an important relationship between the ratio of PS to PMMA in the acrylic copolymer-based bone cement, suggesting that careful optimization of this ratio is essential in the design process for achieving desirable nanomechanical properties. Such considerations are crucial for enhancing the performance of bone cement in clinical applications, ensuring its effectiveness and longevity in orthopedic surgeries.

### 3.5. HR-TEM

**Figure 4:**
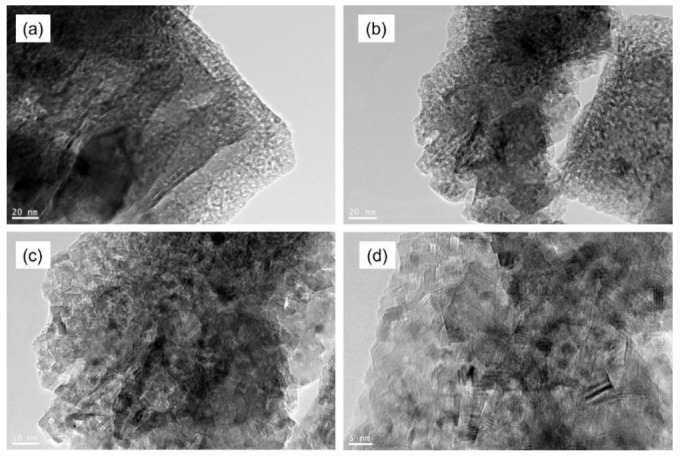
HR-TEM images of (Ag/rGO@TiO_2_)/(PS-PMMA)/PMMA) nanocomposite bone cement.

The morphology of the (Ag/rGO@TiO_2_)/(PS-PMMA)/PMMA nanocomposite bone cement was meticulously examined using high-resolution transmission electron microscopy (HR-TEM). The resulting HR-TEM images of the nanocomposite bone cement samples, presented in Figure 4a–d, clearly showcase the sheet-like structure of reduced graphene oxide (rGO), which is prominently visible throughout the captured images. The darker regions observed in the HR-TEM micrographs represent the Ag and TiO_2_ nanoparticles, highlighting their presence within the composite matrix. Notably, the HR-TEM images reveal excellent dispersion of these nanofillers throughout the polymer matrices, indicating a high degree of homogeneity. This uniform distribution can be attributed to the strong Van der Waals interactions between the nanofillers and the polymer chains, which facilitate effective integration of the components within the composite structure. Such interactions are crucial in enhancing the mechanical and physical properties of the bone cement, thereby improving its suitability for biomedical applications.

### 3.6. In Vitro Studies

**Figure 5:**
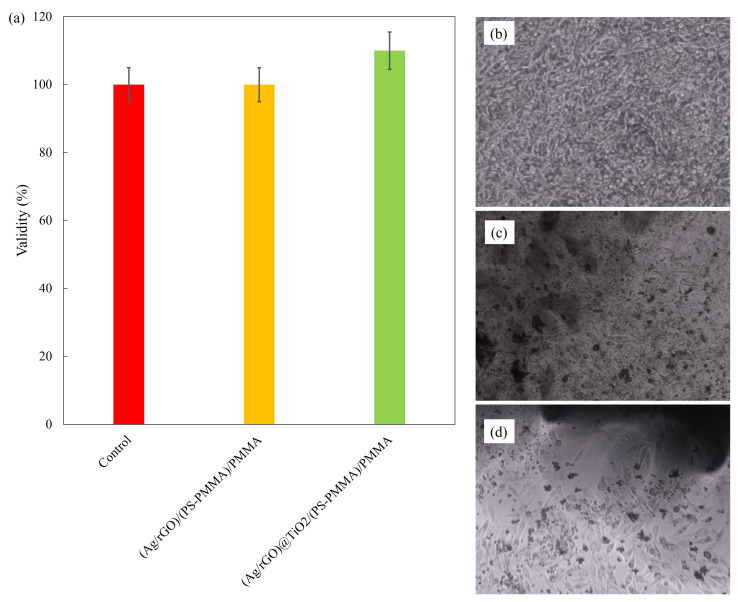
(**a**) Cell validity; (**b**) cell adhesion on control; (**c**) cell adhesion on (Ag/rGO)/(PS-PMMA)/PMMA; and (**d**) cell adhesion on (Ag/rGO)@TiO_2_/(PS-PMMA)/PMMA.

The results of the cell viability assessments are presented in Figure 5a, comparing the control sample with (Ag/rGO)/(PS-PMMA)/PMMA and (Ag/rGO@TiO_2_)/(PS-PMMA)/PMMA. The cell viability data reveal that all tested samples maintained a viability rate equivalent to that of the control group, measuring 100%. This indicates that the cytotoxic effects associated with both the (Ag/rGO)/(PS-PMMA)/PMMA and (Ag/rGO@TiO_2_)/(PS-PMMA)/PMMA formulations are minimal, suggesting their safety for biological applications. Figure 5b–d illustrates the microscopic images of MG63 cell adhesion on the control and (Ag/rGO)/(PS-PMMA)/PMMA and (Ag/rGO@TiO_2_)/(PS-PMMA)/PMMA, respectively. Figure 5b presents an analysis of the MG-63 cells under control conditions. In this depiction, the morphology of the MG-63 osteosarcoma cell line is illustrated, highlighting its characteristic spindle-shaped appearance and adhesion properties. The cells exhibit a uniform distribution, indicating a healthy growth pattern without any significant alterations or stress markers. This control group serves as a baseline for comparing the effects of experimental treatments on cell behavior and viability. The cellular arrangement and density provide essential insights into the overall health and functionality of the MG-63 cells in a standard culture environment. Figure 5c,d shows the MG-63 cell growth in the presence of the bone cement samples. The dark sheet-like and spherical regions observed in Figure 5c and the dark cluster-like areas in Figure 5d are related to the presence of the respective nanofillers within the copolymer matrix. The analysis of cell adhesion demonstrates that the inclusion of the Ag/rGO nanofiller within the acrylic bone cement did not significantly impede cell growth on the polymer surfaces. In contrast, a notable enhancement in cell adhesion was observed when the Ag/rGO@TiO_2_ nanofiller was incorporated into the bone cement formulation. This suggests that the dual-filler system may provide additional benefits for promoting cellular interactions, which could be advantageous for applications in regenerative medicine and tissue engineering.

## 4. Conclusions

This study investigates the impact of a silver nanoparticle/reduced graphene oxide@titanium dioxide nanocomposite (Ag/rGO@TiO_2_) on the mechanical properties and biocompatibility of poly(styrene-co-methylmethacrylate/polymethyl methacrylate) (PS-PMMA/PMMA)-based bone cement, in comparison to commercial PMMA/MMA bone cements. The chemical, structural, and mechanical properties of the Ag/rGO@TiO_2_ nanocomposites when integrated into PS-PMMA bone cement ((Ag/rGO@TiO_2_)/(PS-PMMA)/PMMA) were thoroughly evaluated through a series of analytical techniques. The results indicated an enhancement in the elastic modulus (E) of the bone cement through the integration of Ag/rGO@TiO_2_ nanocomposites, achieving a value of 5.09 GPa. In contrast, the commercial bone cements comprising PMMA/MMA displayed lower elastic modulus values, ranging between 1.7 GPa and 3.7 GPa [60]. This increase in the elastic modulus of the modified bone cement suggests its potential for load-bearing conditions, thereby indicating good durability and strength in practical applications. At the same time, a decrease in the hardness value was noted for Ag/rGO@TiO_2_)/(PS-PMMA)/PMMA compared to PMMA/MMA bone cement, which is related to the possible interaction of 2D-rGO nanosheets with the polymeric chains. In vitro biocompatibility assessments demonstrated that the modified bone cement was non-toxic to the MG63 osteosarcoma cell line, suggesting that it could provide a safe and effective option for clinical applications in bone repair and regeneration. These findings underscore the promising potential of Ag/rGO@TiO_2_ nanocomposites in enhancing the mechanical and biocompatibility profiles of PS-PMMA/PMMA-based bone cement.

## Data Availability

The raw data supporting the conclusions of this article will be made available by the authors on request.
